# Outbreak of multiple strains of non-O157 Shiga toxin-producing and enteropathogenic *Escherichia coli* associated with rocket salad, Finland, autumn 2016

**DOI:** 10.2807/1560-7917.ES.2018.23.35.1700666

**Published:** 2018-08-30

**Authors:** Sohvi Kinnula, Kaisa Hemminki, Hannele Kotilainen, Eeva Ruotsalainen, Eveliina Tarkka, Saara Salmenlinna, Saija Hallanvuo, Elina Leinonen, Ollgren Jukka, Ruska Rimhanen-Finne

**Affiliations:** 1 National Institute for Health and Welfare, Health Security Department, Helsinki, Finland; 2 Environmental Health, Social and Health Services Espoo, Espoo, Finland; 3 City of Helsinki, Communicable Diseases, Epidemiology Unit, Social Services and Health Care Sector, Helsinki, Finland; 4 Division of Infectious Diseases, Inflammation Center, Helsinki University Central Hospital, Helsinki, Finland; 5 Department of Clinical Microbiology, University of Helsinki and Helsinki University Hospital, HUSLAB, Helsinki, Finland; 6 Finnish Food Safety Authority Evira, Research and Laboratory Services Department, Microbiology Research Unit, Helsinki, Finland; 7 Finnish Food Safety Authority Evira, Food Safety Department, Microbiological Food Safety Unit, Helsinki, Finland

**Keywords:** enteropathogenic E. coli, EPEC, epidemiology, food-borne infections, gastrointestinal disease, outbreaks, Shiga toxin-producing E. coli, STEC

## Abstract

In August 2016, an outbreak of Shiga toxin-producing *Escherichia coli* (STEC) and enteropathogenic *E. coli* (EPEC) with 237 cases occurred in the Helsinki metropolitan area, Finland. Gastroenteritis cases were reported at 11 events served by one catering company. Microbiological and epidemiological investigations suggested rocket salad as the cause of the outbreak. STEC ONT:H11 and EPEC O111:H8 strains isolated from food samples containing rocket were identical to the patient isolates. In this outbreak, the reported symptoms were milder than considered before for STEC infection, and the guidelines for STEC control measures need to be updated based on the severity of the illness. Based on our experience in this outbreak, national surveillance criteria for STEC have been updated to meet the practice in reporting laboratories covering both PCR-positive and culture-confirmed findings. We suggest that EPEC could be added to the national surveillance since diagnostics for EPEC are routinely done in clinical laboratories.

## Background

*Escherichia coli* are Gram-negative rod-shaped bacteria and part of the normal bacterial flora in the gastrointestinal tract, while diarrhoeagenic *E. coli* pathotypes such as Shiga toxin-producing *E. coli* (STEC) and enteropathogenic *E. coli* (EPEC) are able to cause gastrointestinal infections [1]. STEC can lead to a severe disease, such as haemolytic-uraemic syndrome (HUS) [2]. The risk of HUS has been related especially to children under 5 years and to elderly people. HUS is characterised by acute onset of microangiopathic haemolytic anaemia, renal injury and low platelet count.

More than 400 STEC serotypes have been recognised, of which the best-known serotype is O157:H7 [1]. The most common non-O157:H7 serotypes causing human infections are O26, O103, O111 and O145 [3]. The virulence of STEC is largely based on the production of Shiga toxin 1 or 2 and is identified by detecting the presence of *stx*1 or *stx*2 genes [1,4]. The virulence of EPEC is caused by its capability to form attaching and effacing (A/E) lesions in the small intestine. This capability requires the presence of virulence genes called the locus of enterocyte effacement (LEE) in a pathogenity island (PAI) that encodes intimin [4]. Unlike STEC, EPEC do not produce Shiga toxin. EPEC are divided into two distinct groups by the presence of EPEC adherence factor plasmid (pEAF) expressing bundle-forming pili (BFP), which is a virulence determinant of typical EPEC (tEPEC) [5]. Thus atypical EPEC (aEPEC) are defined as *E. coli* that produce A/E lesions but do not express BFP. Typical EPEC are best known as a cause of infantile diarrhoea, especially in developing countries [6]. Diarrhoea-causing aEPEC have been shown to be separate group without a close relation to tEPEC, but some serotypes are genetically related to STEC [5]. The pathogenity of aEPEC has been questioned but their involvement with diarrhoeal outbreaks supports the idea that certain strains are diarrhoeagenic [1,7].

Both STEC and EPEC are transmitted through the faecal-oral route, and outbreaks caused by STEC and aEPEC have been described after ingestion of contaminated food or water [7,8]. STEC is common in ruminants and can be found in foods contaminated by ruminant faeces [9]. Most studies on STEC have focused on the serotype O157:H7, but infections and outbreaks caused by non-O157 strains are increasingly reported in Europe and elsewhere [10-13]. Atypical EPEC strains are found in animals used for food production, such as cattle, sheep, goat, pig and poultry, in contrast to tEPEC that has been found only in humans [1,14].

Since 1995, clinicians and clinical microbiology laboratories have been obliged to report culture-confirmed STEC infections to the Finnish Infectious Disease Registry (FIDR) maintained by the National Institute for Health and Welfare (THL) in Finland. EPEC infections are not reportable. Since PCR instead of culture became the standard for screening of diarrhoeal patients in 2013, the incidence of reported STEC infections has increased in Finland to 1.2–1.8 per 100,000 population between 2013 and 2015 compared with 0.2–0.6 per 100,000 between 2000 and 2012. From 1997 to 2015, six food- or waterborne STEC outbreaks were detected in Finland (Table 1).

**Table 1:** Food- and waterborne STEC outbreaks, Finland, 1997–2015

Year	Number of cases	Serotype	Suspected/confirmed origin
1997	14	STEC O157:H7	Swimming water [32]
1998	5	Not known	Hamburger restaurant [34]
2001	4	STEC O157:H7	Kebab meat [34]
2012	11	STEC O157:H7	Unpasteurised milk, cattle [18]
2013	17	STEC O157:H7	Meal from an institutional kitchen [27]
2013	16	STEC O157:H7	Unspecified, widely sold product [27]

STEC: Shiga toxin-producing *Escherichia coli*.

On 23 August 2016, one local environmental health authority in southern Finland notified the National Registry for Food and Waterborne Outbreaks of gastroenteritis after events to which one company had supplied catering during the weekend 19 to 21 August 2016. Based on the symptoms, norovirus was suspected as the cause of illness in the notification. Soon after, EHEC was revealed as the causative agent in microbiological testing.

We investigated the outbreak to identify its extent and source together with the regional and local health and environmental health officials in affected municipalities in the Hospital District of Helsinki and Uusimaa in order to prevent further cases and outbreaks.

## Methods

### Epidemiological investigation

Twelve events were organised and catered by one company from 19 to 21 August 2016 in the Helsinki metropolitan area. Lists with name, age and place of residency of the participants were supplied to local health officials by the contact persons of each event. The number of exposed people in this outbreak was based on these lists.

We conducted a retrospective cohort study to gather information on demographic and clinical characteristics of the subjects, and food exposure from 11 events for which gastroenteritis cases had been reported. Respondents were invited by email or phone to reply using a web-based questionnaire; the link was sent to them by the organisers of each event within 8 days of the notification of the outbreak to the authorities.

We defined a case as a person with a stool sample positive for STEC or EPEC between 19 August and 22 September 2016, or with symptoms of diarrhoea more than three times a day between 20 August and 3 September 2016, who had participated in one of the events that the catering company had served between 19 and 21 of August 2016.

We calculated the incubation period and the duration of the illness, and risk ratios (RR) with 95% confidence intervals (CI) to explore the associations between single and pooled exposures and the outcome. Pooled exposures included foods that shared the same ingredients, such as food containing rocket (chicken fillet in oil with fresh herbs, pesto marinated chicken fillet and thyme marinated roast beef garnished with rocket), cheesecakes (mango cheesecake, raspberry white chocolate cake and chocolate–orange cake) and salmon (lime spiced salmon, wine–lemon marinated salmon, smoked salmon and Caesar salad with smoked salmon). For all analyses, a p value < 0.05 was considered significant. The STATA Data Analysis and Statistical Software version 14 (Stata Corporation, College Station, United States (US)) was used to perform the analysis.

### Environmental and microbiological investigation

The local environmental health authority inspected the premises of the catering company on 24 August and collected menu lists of each event. Food samples were taken for microbiological analysis. All three staff members in the catering company were interviewed and faecal samples were advised by the local environmental health authority to be collected from the staff members. When rocket was identified as a possible source of the outbreak, the municipal authority contacted the importer in order to identify when, where and how much of that batch of rocket was distributed in Finland.

Stool samples from the event participants and staff members were analysed at two clinical microbiology laboratories using faecal culture for *Salmonella*, *Shigella*, *Yersinia*, *Campylobacter*, *Clostridium perfringens*, *Bacillus cereus* and *Staphylococcus aureus* and stool PCR for *Salmonella*, *Yersinia*, *Campylobacter*, *Vibrio cholerae*, *Shigella*, norovirus, STEC, EPEC, enterotoxigenic *E.coli* (ETEC) and enteroaggregative *E. coli* (EAEC) [15-17]. PCR-positive STEC samples were cultured and the isolates from positive cultures were sent to THL for typing. The detection of the virulence genes *stx*1, *stx*2, *eae*, *hly*A, and *saa* by PCR was performed as described previously [18]. For determination of O-serogroup, the group antiserum test and the latex agglutination tests for the most common serogroups were used as first-line method before proceeding to conventional serotyping [18]. The virulence genes and O:H serotype were also examined by whole genome sequencing. The genomic libraries were prepared with the Nextera XT Sample Preparation and Index Kits (Illumina, SanDiego, US) and run by MiSeq (Illumina) sequencer with 150 bp pair-end reads. The FASTQ sequences were assembled using Velvet in Ridom SeqSphere (Ridom GmpH, Münster, Germany) and the FASTA files were submitted to the Centre for Genomic Epidemiology (CGE; Lyngby, Denmark) to identify virulence genes [19], O:H serotype [20] and multilocus sequence type (MLST) [21].

All STEC-positive findings from 21 August (date for the first outbreak-related STEC case reported to health authorities) to 22 September reported to FIDR were linked to the names, ages and places of residency on the participant lists of the 11 events with reported gastroenteritis cases, and to the interviews of STEC-positive persons done by the local infectious diseases nurses. As EPEC-positive findings are not routinely reported to THL, we went through all EPEC-positive findings from the period 19 August to 22 September in the main laboratory of the Helsinki metropolitan area (HUSLAB) that serves the southern part of Finland where outbreak took place. The personal details (name and age) of EPEC cases were linked to the participant lists of the events to find out if the EPEC-positive cases were related to this outbreak. In addition, we went through the personal details of all cases whose stool samples were tested with stool PCR or tested for STEC specifically. These personal details were then linked to the lists of participants and to those who had answered the study questionnaires.

Food samples were collected from the catering company and from the customers and investigated by generic *E. coli* enumeration at MetropoliLab Oy or for STEC and EPEC with real-time PCR and culture at Finnish Food Safety Authority Evira [22]. For isolation, samples of 25 g were enriched in buffered peptone water (BPW) (Merck, Darmstadt, Germany) for 18–24 h. Enrichments were diluted and 0.1 mL aliquots from dilutions 10^−3^ to 10^−8^ were spread on parallel plates of Harlequin SMAC-BCIG (cefixime-tellurite sorbitol MacConkey agar with 5-bromo-4-chloro-3-indoxyl-β-D-glucuronide, Lab M, Lancashire, United Kingdom (UK)), CHROMagar STEC (CHROMagar, Paris, France), SHIBAM (BAM Media M195 [23]) and TBX agar (HarlequinTM TBGA, Tryptone Bile Glucuronide Agar, Laboratory M, Lancashire, UK). The plates were incubated at 37 °C for 18–24 h and typical colonies were confirmed by real-time PCR for the presence of *stx*1 and/or *stx*2 (*stx* 1/2) and *eae* genes (iQ-Check STEC VirX PCR Detection Kit, Bio-Rad Laboratories, Hercules, US) or *stx*1, *stx*2 and *eae* genes (TaqMan Assay, ISO, Thermo Fisher Scientific, Life Technologies, Carlsbad, US). STEC and EPEC isolates from the food were compared with patient samples by genotyping with pulsed-field gel electrophoresis (PFGE) with *Xba*I digestion according to the PulseNet protocol [24].

## Results

### Descriptive and analytical epidemiology

Participants with gastroenteritis were reported in 11 of 12 events held between 19 and 21 August. The number of participants in the 11 events held in five municipalities was 670 (16 to 113 per event). In total, 427 of 670 participants (64%) took part in the retrospective cohort study. Of the respondents, 237 (56%) fulfilled the case definition (Figure). Their median age was 31 years (interquartile range (IQR): 30–35; range: 22–36) and 134 (57%) were female. The median incubation period of illness was 19 h (IQR: 16–24; range: 8–112) and the median duration of symptoms was 45 h (IQR: 24–66; range: 1–288).

**Figure:**
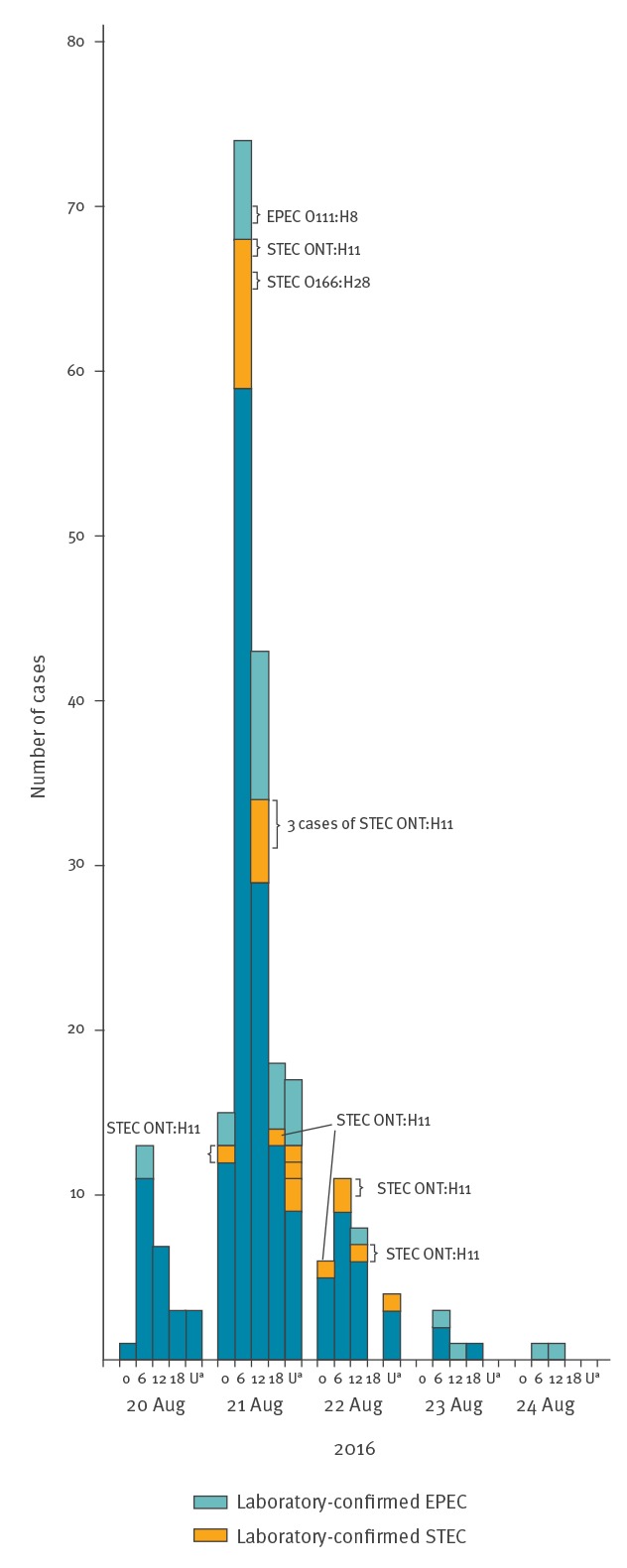
Number of gastroenteritis cases by date of symptom onset, Finland, 20–24 August 2016 (n = 231)

EPEC: Shiga toxin-producing *Escherichia coli*; STEC: enteropathogenic *E. coli*; U: unknown.

Serotypes for typed cases are shown. The onset of symptoms is shown by date in 6 h intervals starting at midnight. U stands for unknown time within that day.

Forty-two EPEC cases and 22 STEC cases answered the retrospective cohort study. Diarrhoea and stomach pain were their most common symptoms. One of the STEC cases had bloody diarrhoea (Table 2). EPEC cases reported less often symptoms than STEC cases (Table 2). None of the patients had HUS.

**Table 2:** Characteristics of STEC and EPEC infections among those who answered the questionnaire study, Finland, August 2016 (n = 64)

	STEC infection (n = 22)		EPEC infection (n = 42)	
	n	%	n	%
Female sex	11	50	30	71
Median age in years (range)	34 (7–63)		31 (1–80)	
**Frequency of symptoms**				
Asymptomatic	0	0	12	29
Diarrhoea	19	86	21	50
Bloody diarrhoea^a^	1	11	0	0
Vomiting	3	14	5	12
Stomach pain	18	82	27	64
Fever	3	14	10	24
Chills	8	36	10	24
Headache	7	32	14	33
Visited healthcare facilities	10	45	23	55
Incubation period in hours (median)^b^	20 (13–44)		23 (13–112)	
Duration of symptoms in hours (median)^c^	52 (16–108)		48 (1–156)	

EPEC: Shiga toxin-producing *Escherichia coli*; STEC: enteropathogenic *E. coli*.

^a^n = 9 for STEC and n = 27 for EPEC infection.

^b^n = 21 for STEC and n = 27 for EPEC infection.

^c^n = 21 for STEC and n = 21 for EPEC infection.

Two of three staff members of the catering company reported mild diarrhoea starting on 21 August and one was asymptomatic. None of them was working while symptomatic but all had eaten rocket-containing salad during the preparation of the catered meals on 20 August. One of the staff members had STEC and two (one asymptomatic) had EPEC infections. STEC was positive in culture, but could not be isolated for serotyping. EPEC infections were diagnosed with PCR.

In 11 events, 50 different dishes and bread, water, milk, juice, tea and coffee were served (five to 16 dishes per event). In univariate analysis of each event, six food items served at four events were significantly associated with the illness (Table 3). Of these, only chicken fillet in oil with fresh herbs and lime spiced salmon contained ingredients that were common to foods served at several events. In pooled analysis, only food containing rocket was significantly associated with gastroenteritis (RR = 1.93; 95% CI: 1.38–2.70; p value < 0.001). In univariate analysis of different events, food containing rocket was associated with gastroenteritis with the RR varying from 0.85 (95% CI: 0.72–1.00; p value 1.000) to 7.67 (95% CI: 1.18–49.74; p value < 0.001).

**Table 3:** Association of dishes with gastroenteritis in univariate analysis, Finland, August 2016 (n = 427)

Exposure	Cases exposed		Controls exposed		Risk ratio (95% CI)	p value
	n/N	%	n/N	%		
Rustic bread	11/11	100	7/14	50	2.00 (1.18–3.38)	0.008
Lettuce with melon	27/34	79	1/5	20	3.97 (0.68–23.11)	0.017
Chicken fillet in oil with fresh herbs including rocket^a^	25/32	78	1/5	20	3.91 (0.67–22.76)	0.021
Chicken fillet in oil with fresh herbs including rocket^a^	23/30	76	1/10	10	7.67 (1.18–49.7)	< 0.001
Lime raw spiced salmon	21/31	67	2/9	22	3.05 (0.88–10.60)	0.023
Hummus	12/21	57	3/15	20	2.86 (0.97–8.39)	0.041

^a^Served at different event sites.

Only statistically significant associations are shown.

### Microbiological investigation

Between 19 August and 2 October 2016, 31 samples were positive for STEC and 62 for EPEC by PCR in event participants and staff members. At least three people had both STEC and EPEC as they were EPEC-positive in a control sample that was taken because of primary STEC infection. Most of the PCR findings were weak signals and 11 STEC isolates could be cultured for typing: 10 identical STEC ONT:H11 strains (ST295, *stx*2a+ , *saa*+ , *eae*−, *hly*A+ , sorbitol+; ENA run accession number: ERR2438124) and one STEC O166:H28 strain (ST1819, *stx*2b+ , *saa*−, *eae*−, *hly*A+ , sorbitol+; ENA run accession number: ERR2438125). In addition, five samples with the strongest signals for EPEC were cultivated for isolation of EPEC. Among them, there were three different strains: two isolates were EPEC O111:H8 (ST327, *saa*−, *stx*−, *eae*+ , *hly*A−, sorbitol+; ENA run accession number: ERR2438126), two were EPEC O171:H25 (ST5683, *saa*−, *stx*−, *eae*+ , *hly*A−, sorbitol+; ENA run accession number: ERR2438127) and one was EPEC ONT:H21 (ST40, *saa*−, *stx*− *eae*+ , *hly*A−, sorbitol+; ENA run accession number: ERR2438128). Twenty-three culture-positive STEC cases were reported to the FIDR.

In the preliminary analysis of food samples (parsley, tomato, mixed salad, melons, lamb’s lettuce, smoked salmon, rocket (unopened package), two different samples of chicken fillet in oil with fresh herbs including rocket and thyme-marinated roast beef garnished with rocket), elevated *E. coli* levels (3,100 cfu/g) were found in rocket. Samples of chicken fillet in oil with fresh herbs including rocket and of thyme-marinated roast beef garnished with rocket were *stx*2- and *eae*-positive. STEC and EPEC strains could be isolated from SHIBAM and TBX agars, the more selective agars did not support their growth. In further examinations by PFGE, STEC strains isolated from samples of chicken fillet in oil with fresh herbs including rocket and from thyme-marinated roast beef garnished with rocket were found to be identical to the STEC ONT:H11 patient isolates. EPEC O111:H8 identical to the human isolates were found in these same samples that contained unheated rocket. One of the five samples of rocket in an unopened package was positive for *stx* in PCR and two samples were *eae*-positive. EPEC strains could not be isolated from the unopened package of rocket. In one sample, STEC was detected on primary plating, but the strain could not be purified for further investigations.

### Traceback investigations and public health measures

The premises of the catering company were inspected on 24 August and nothing remarkable was found in the inspection.

Within 8 days of the outbreak notification, information on STEC infection was shared by the regional and local environmental authorities to the event participants with the advice to use proper hand hygiene and to seek medical care and give faecal samples if gastrointestinal symptoms occurred. This was highlighted especially to those working in the food industry, nursing young children or attending child daycare, and to symptomatic children and pregnant women. Symptomatic children, elderly people and pregnant women were advised to contact primary healthcare because of the increased risk of HUS. Information letters were sent within a week after the start of the outbreak. In addition, we asked two to five symptomatic persons from each event to give stool samples to confirm a common pathogen.

The catering company had received 10 200 g packages of fresh rocket on 18 August. The best before date of this rocket was 24 August 2016. The country of origin was Denmark and that of packaging Sweden. Food control authorities in Denmark performed an inspection at the grower of the rocket. According to them, the control documentation of the farmer was satisfactory, including a satisfactory result for laboratory analyses of samples from rocket (indicator bacteria) and for the quality of water used in the plant. Also general maintenance of the production facilities was satisfactory. The manufacturer received rocket from the grower on 13 August. The report provided by the manufacturer showed that the batch was used on 15 August to produce a little over 1,000 kg of packed rocket, of which more than 800 kg was delivered to Finland by several importers. In Finland, the product was distributed further to different parts of the country.

THL together with Evira and the regional and local health authorities announced the outbreak in the media three times between the end of August and early October to inform healthcare professionals and the public. The outbreak was declared over on 5 October 2016. Rapid Alert System for Food and Feed (RASFF) notification [25] was published 5 September 2016 and further updated 22 September and 12 October.

## Discussion

We described an outbreak where over 200 persons fell ill with gastroenteritis after eating fresh rocket. The majority of laboratory-tested persons had STEC or EPEC in stools. The rocket was served at 11 events catered by one company and was found contaminated by *E.coli*. *Stx* and *eae* genes were detected in rocket sampled from an unopened package, and identical STEC and EPEC strains were isolated in rocket-containing dishes and patients´ samples. At the time of the outbreak investigation, no rocket of the contaminated batch was on the market anymore. Two staff members of the catering company had gastroenteritis symptoms at the same time as the event participants. They had eaten rocket during the preparation of the dishes and had not been working while symptomatic.

This outbreak affecting the Helsinki metropolitan area was large for Finnish standards. Rocket was confirmed as the cause of the outbreak in the microbiological analyses of food samples and human samples as well as in the questionnaire study among the exposed. Since 1997, six STEC outbreaks with a median of 13 cases have been reported in Finland (Table 1). Food-related outbreaks caused by EPEC are seldom reported. An outbreak with over 600 cases caused by EPEC O111 was detected in 1990s in a school in Finland, however, EPEC was not found in any tested food items [7]. In Norway, a nursery outbreak after a farm visit was caused by STEC O26 and EPEC O76 and the animals were found to be the reservoir of those pathogens [8]

The outbreak was investigated jointly by three municipal outbreak investigation groups in the Helsinki metropolitan area, the Hospital District of Helsinki and Uusimaa, THL and Evira. The identification of STEC was communicated rapidly from HUSLAB to the epidemiologists at the Hospital District of Helsinki and Uusimaa and Helsinki city, and from them to the municipal outbreak investigation group that had started the outbreak investigation. When STEC was found in stools from the event participants, the regional and local investigation groups requested THL to coordinate the investigation. The rapid diagnostics and information flow allowed us to search and contact participants belonging to STEC risk groups (children, pregnant women, elderly people and those working in the food industry or caring for children under school age) [26]. Active contacting of risk groups was discontinued when it was found that no severe manifestations of STEC infections were reported.

In this outbreak, the diagnostic identification of STEC and EPEC was based on a stool PCR test that is faster and more sensitive than culture [15]. Testing of STEC increased in Finland after the introduction of PCR screening tests for gastroenteritis and is done also for indications other than bloody diarrhoea [15]. Therefore, more and milder STEC infections have been reported since 2012 [27-29]. In this outbreak, all STEC cases had mild gastrointestinal symptoms, even though the isolated strains had the *stx*2a gene, which has been associated with HUS [30,31]. The absence of severe symptoms may be explained by the lack of the *eae* gene, which has also been associated with HUS [31]. Also, only few children, who are in a greater risk for HUS, were among the exposed [13]. The incubation period for STEC infection was shorter than usually described 3 to 4 days, as the median being 20 hours in this outbreak [32,33]. For this reason and based on the mild symptoms, norovirus was first suspected as the cause of illness before STEC was revealed in the PCR screening tests. The variation in symptom severity in STEC infection is wider than considered at the time the Finnish national guidelines for STEC control measures were compiled, and they need to be updated accordingly [33]. There is a need to determine EHEC control measures based on the HUS risk profile of the STEC strain.

During the outbreak, the clinical laboratory staff noticed an increase in EPEC findings connected to outbreak samples. Even though almost one third of EPEC infections were asymptomatic, we suggest that EPEC was a significant, symptom-causing pathogen in this outbreak. Three different EPEC serotypes were isolated among the cases, but there may have been more serotypes since isolation was performed only for the five samples with the strongest PCR signals. Of these cases, three replied to the questionnaire. One with serotype O111:H8 reported stomach pain and fever, and two were asymptomatic (one with serotype O111:H8 and one with O171:H25). Atypical EPEC has been found to be more closely related to STEC than to typical EPEC, and it has been suggested that aEPEC may derive from STEC that has lost toxin-producing capability [5,6]. In this outbreak, however, the EPEC serotypes found in patients were not similar to the STEC serotypes in the outbreak.

Three STEC cases were positive for EPEC in a control sample. It is probable that the true number of mixed infections was higher since the stool PCR test is not able to differentiate STEC and EPEC in concomitant infections [32] and only STEC is reported as a positive result. The probability of mixed infections is also supported by the fact that two different strains of STEC and three different strains of EPEC were found in the patients, and both STEC and EPEC were found in the food samples.

In the cohort study, food containing rocket was found significantly associated with gastroenteritis. This was supported by microbiological results from patient samples and food samples showing identical STEC and EPEC strains. Rocket from the same batch was also delivered to other parts of Finland, but no other STEC cases with the same serotypes were identified. After rocket was communicated in the media as the source of the outbreak, some patients’ faecal samples revealing EPEC were sent to HUSLAB with a note of recent rocket consumption but without a connection to the eleven events. It is also probable that persons with mild gastroenteritis did not seek medical care and therefore possible cases were not identified.

In Finland, the FIDR reporting criteria included only culture-confirmed STEC, although the clinical laboratories have used PCR since 2012. Culture confirmation criteria, however, can be interpreted as isolation of an STEC strain or growth in a mixed culture from which isolation cannot be done. In Finland, the clinical microbiology laboratories are requested to submit all STEC isolates to THL for confirmation and genotyping. From 2010 to 2012, STEC isolate was confirmed at THL in 97% of FIDR notifications (data not shown). From 2013 to 2016, the proportion of successful STEC isolations among all STEC findings decreased from 93% to 64%. In the outbreak described here, 35% of the patients’ STEC strains could be isolated from cultures. At the beginning of the investigation, FIDR could not be used for outbreak detection and investigation purposes because of more than 1 week reporting delay. Based on our experience in this outbreak, national STEC surveillance criteria have now been updated to meet the practice in reporting laboratories. We suggest that even EPEC diagnostics, which are routinely done in clinical laboratories, could be added to the FIDR surveillance.
